# Supplementary material for: Ionization and structural properties of mRNA lipid nanoparticles influence expression in intramuscular and intravascular administration
Source: Commun Biol. 2021 Aug 11;4:956. doi: 10.1038/s42003-021-02441-2 (PMC8358000; doi:10.1038/s42003-021-02441-2)
Supplement: Supplementary file 2 — Supplementary Information [file 42003_2021_2441_MOESM2_ESM.pdf]

Ionization and Structural Properties of mRNA Lipid Nanoparticles that Influence Expression in Intramuscular and Intravascular Administration

Manuel J. Carrasco[1], Suman Alishetty[1], Mohamad-Gabriel Alameh[2], Hooda Said[1], Lacey Wright[1], Mikell Paige[3], Ousamah Soliman[2], Drew Weissman[4], Thomas E. Cleveland IV[5], Alexander Grishaev[5], Michael D. Buschmann[1]*

[1] Department of Bioengineering, George Mason University

4400 University Drive, MS 1J7, Fairfax, VA 22030

[2] Perelman School of Medicine, University of Pennsylvania,

130 Stemmler Hall, 3450 Hamilton Walk, Philadelphia, PA 19104

[3] Department of Chemistry & Biochemistry, George Mason University

4400 University Drive, Fairfax, VA 22030

[4]Perelman School of Medicine, University of Pennsylvania

410B Hill Pavilion, 380 S. University Ave, Philadelphia, PA 19104

[5] Institute for Bioscience and Biotechnology Research

National Institute of Standards and Technology

9600 Gudelsky Dr., Rockville, MD 20850

*Correspondence:

Michael D. Buschmann, Chair in the Bioengineering Department, George Mason University, 4400 University Drive, MS 1J7, Fairfax, VA 22030, United States. E-mail: mbuschma@gmu.edu

List of Supplementary Figures and Tables

**Figure S1.** ^{1}H NMR spectra for water-soluble analogues of ionizable lipids to measure pKa in aqueous media
**Table S1.** Published TNS assays using different concentrations of ionizable lipid and TNS
**Figure S2.** Comparison of pKa obtained by TNS using assay conditions from three different publications
**Table S2.** Comparison of pKa obtained by TNS using assay conditions from 3 different publications and published pKa values for LNPs containing these ionizable lipids
**Figure S3.** LNP pKa obtained from the TNS assay using assay conditions from three different publications for 6 ionizable lipids compared to published pKa values for LNPs containing these ionizable lipids
**Figure S4.** Zeta Potential vs pH for Charge Neutral LNPs
**Figure S5.** ^{1}H, ^{13}C and DEPT-NMR Data of Key Compounds

List of Calculations and Derivations

Calculation of pKa of the ionizable lipid in the LNP compared to the pKa of the ionizable lipid in the aqueous phase
Molecular Volume Model of the LNP estimates copies of mRNA per LNP and of the 4 lipid components
Calculation of Elementary Charge and Dielectric Constant of the LNP

Methods and Materials

Materials
Preparation of mRNA Lipid Nanoparticles
TNS Assay
Empty LNP size and zeta potential (ZP) using dynamic light scattering and electrophoretic mobility
Synthesis of water-soluble ionizable lipid analogues
NMR measurement of pKa of water-soluble ionizable lipid analogues

A. DODAP : pKa = 7.65

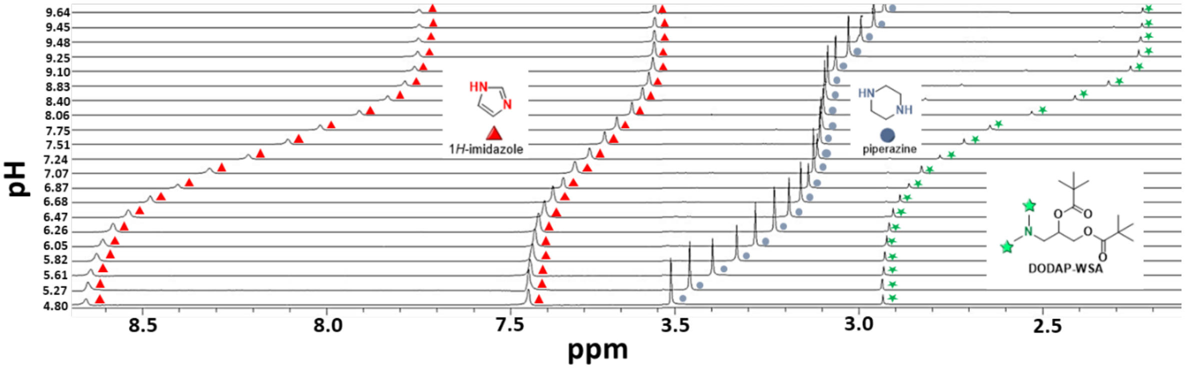

B. DLinDMA, DODMA: pKa= 9.13

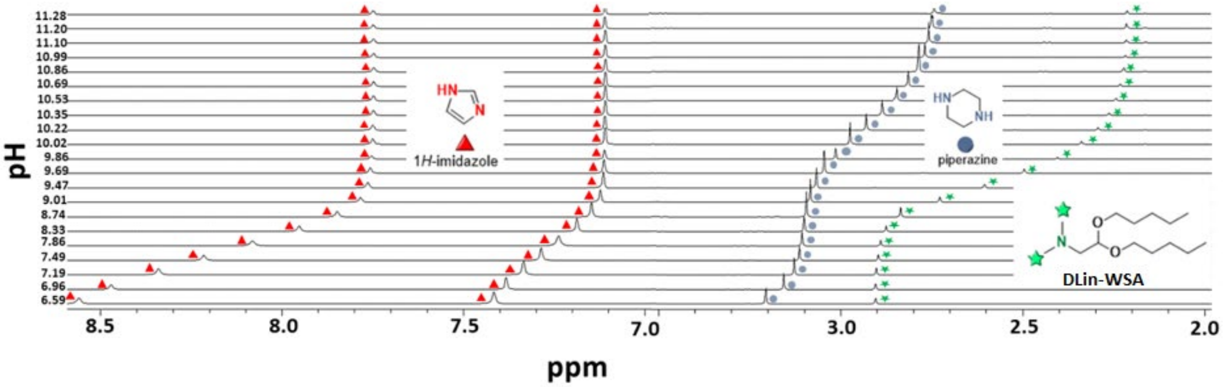

C. DLin-MC3-DMA : pKa = 9.47

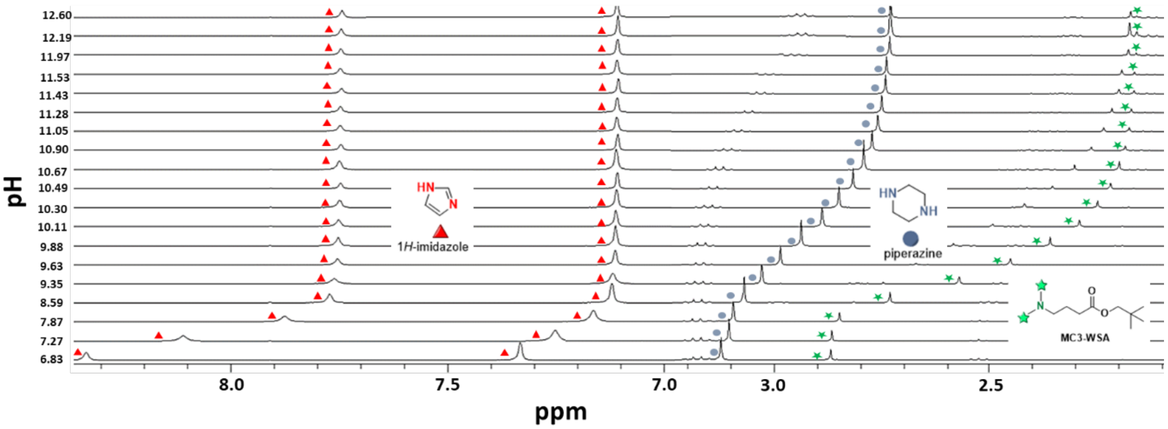

D. DLin-KC2-DMA : pKa = 9.34

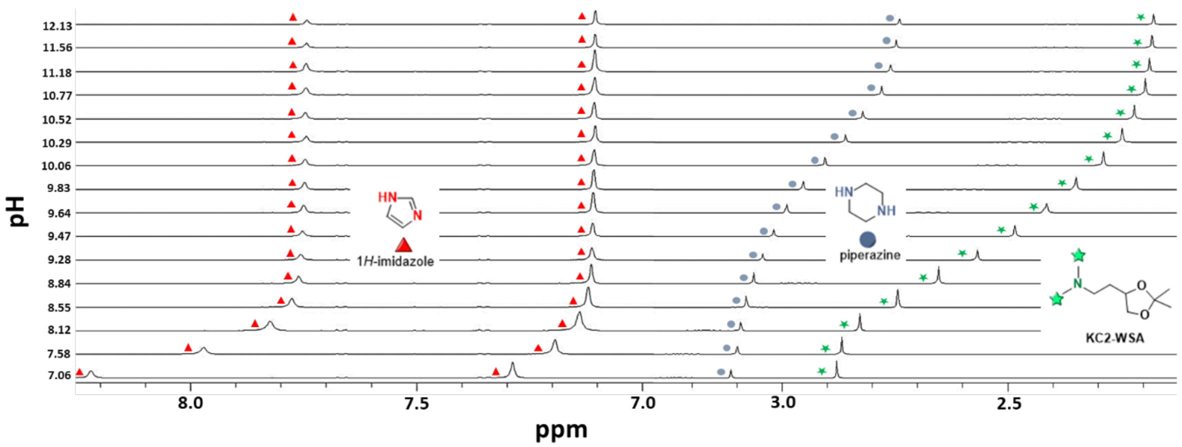

Supplementary Figure 1: ^{1}H NMR spectra for water-soluble analogues of ionizable lipids to measure pKa in aqueous media. Water-soluble analogues of five ionizable lipids were synthesized by replacing the long alkyl chains by shorter less hydrophobic moieties: DODAP-WSA, DLin-WSA, MC3-WSA and KC2-WSA. Solutions with a series of pH were prepared containing the ionizable lipid analogues and pH indicators Imidazole and Piperazine. The pH of each solution was calculated using the chemical shift of the indicators (▲ Imidazole and ● Piperazine) and the chemical shift of the terminal dimethyl protons (✶) was fit to the Henderson–Hasselbalch equation to determine the pKa of the dimethylamine moiety[1]. An alkaline degradation (saponification) product was found for the MC3 analogue located downfield from the non-degraded analogue.

Comparison of Alternative TNS Binding Assay Conditions

Reference	Ionizable Lipid Concentration (μM)	TNS reagent Concentration (μM)	TNS: Ionizable Lipid Ratio
Jayarman 2012[2]	40	1	0.025
Semple 2010[3]	40	1	0.025
Zhang 2011[4]	75	6	0.08
Sabnis 2018[5]	24	6.3	0.26
Heyes 2005[6]	6	6	1
Hajj 2019[7]	1005	6	0.006

Supplementary Table 1. Published TNS Binding assays[2,4,5] using different concentrations of ionizable lipid and TNS. Absolute RFUs in the assay are higher when the ionizable lipid concentration or the TNS concentration is higher while pKa found by fitting to the Henderson–Hasselbalch equation differ by less than 0.2 between assays. The TNS binding assay is incapable of detecting ionization below pH 6 probably due to steric hindrance for TNS binding to ionizable lipid within the LNP. Zeta potential measurements detect LNP net charge and can detect LNP ionization down to at least pH 3 (Figures 1C & 5E).

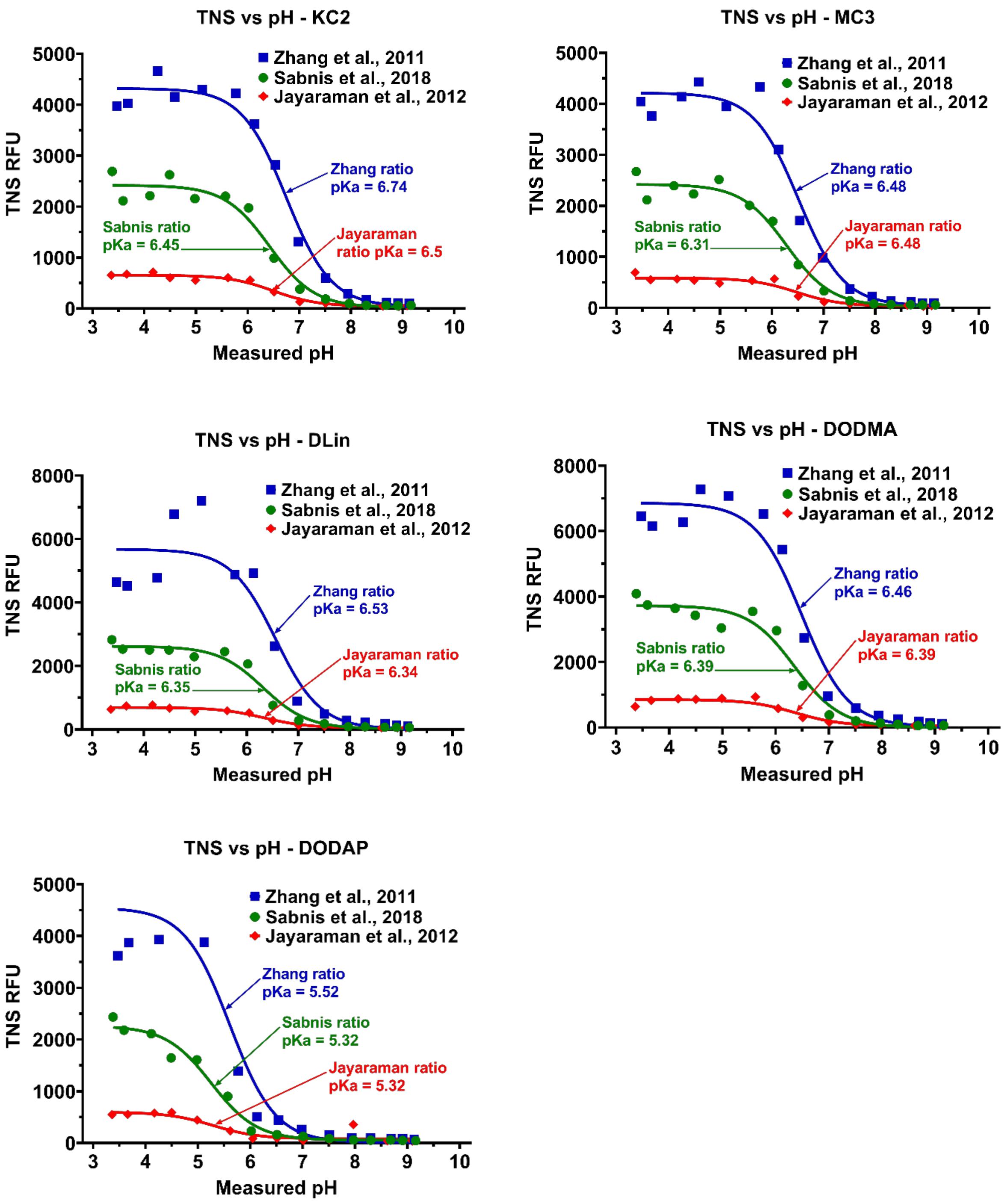

Supplementary Figure 2. Comparison of pKa obtained by TNS using assay conditions from three different publications[2,4,5].

Lipid	Literature pKa	pKa Zhang	pKa Sabnis	pKa Jayaraman
**KC2**	6.68 [2]	6.74	6.45	6.5
**MC3**	6.44 [2]<br>6.3 [5]	6.48	6.31	6.48
**DLin**	6.7 [6]<br>6.8 [3]	6.53	6.35	6.34
**DODMA**	7 [6]	6.46	6.39	6.39
**DODAP**	5.8[8] (not TNS)	5.52	5.32	5.32

Supplementary Table 2. Comparison of pKa obtained by TNS using assay conditions from 3 different publications[2,4,5] and published pKa values for LNPs containing these ionizable lipids[2,3,5,6,8]. pKa using the Zhang method appear to be ~0.18 units higher than by Sabnis.

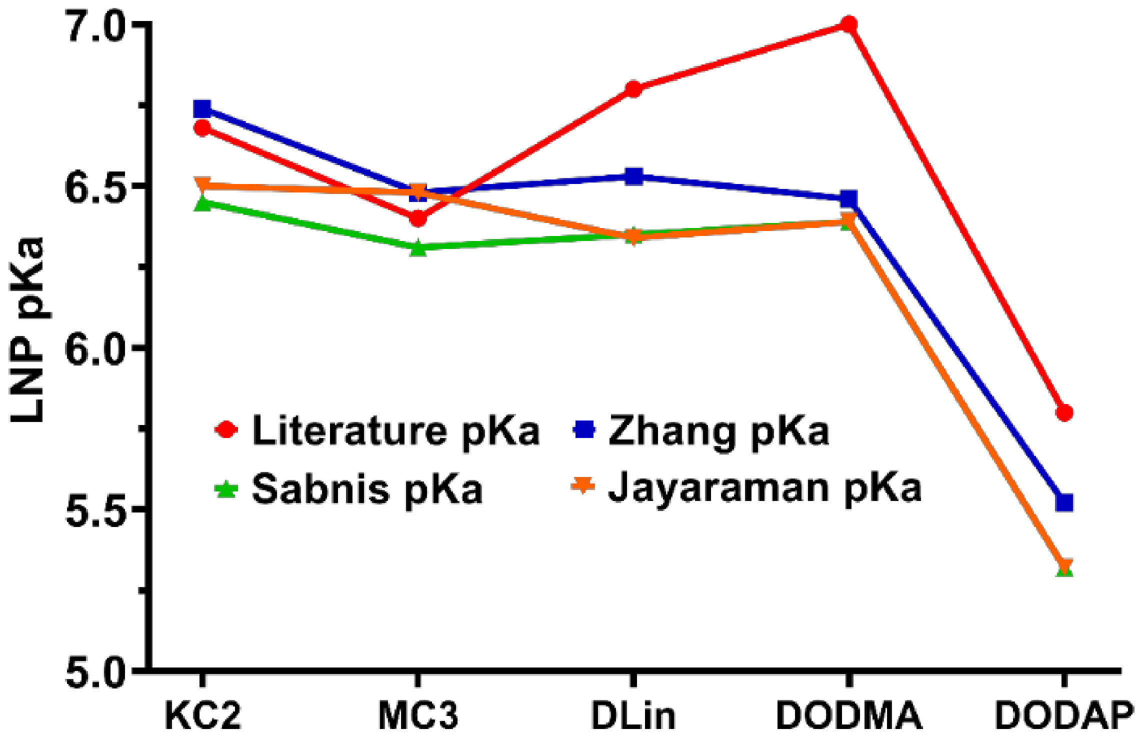

Supplementary Figure 3. LNP pKa obtained from the TNS binding assay using assay conditions from three different publications[2,4,5] for 6 ionizable lipids compared to published pKa values for LNPs containing these ionizable lipids[2,3,5,6,8].

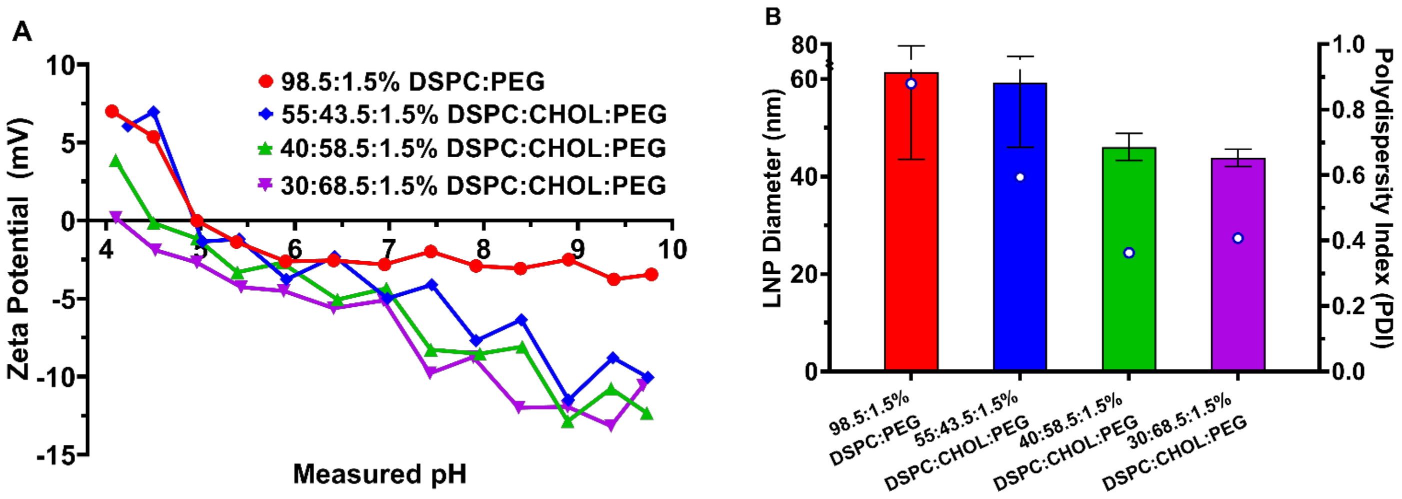

Supplementary Figure 4. Zeta Potential vs pH for Charge Neutral LNPs. Charge neutral LNPs without mRNA were assembled with the lipid mole ratios 30-98.5:43.5-68.5:1.5 (DSPC:Cholesterol:PEG-DMG2000) and using 25 mM sodium acetate buffer pH4 alone to replace the mRNA solution. A) Zeta Potentials of charge-neutral LNPs containing zwitterionic DSPC were slightly positive at low pH and negative at high pH with a transition pI between pH 4 and 5, similar to what has been seen with zwitterionic supported bilayer membranes[9]. This behaviour is most likely due to absorption of OH^- at higher pH and H_3O^+ at lower pH and is consistent with the net negative zeta potential measured at neutral pH for large charge-neutral unilamellar vesicles prepared from palmitoyloleoylphosphatidylcholine[10]. This pH-dependent ion binding may occur for mRNA LNPs containing ionizable lipids, but mostly likely to a much lesser extent since the net positive charge of LNPs at low pH from the ionizable lipid will inhibit H_3O^+ binding and the net negative charge of LNPs at higher pH from mRNA will inhibit binding of OH^-. B) Diameter and PDI of charge neutral LNPs.

^{1}H NMR of DODAP-WSA

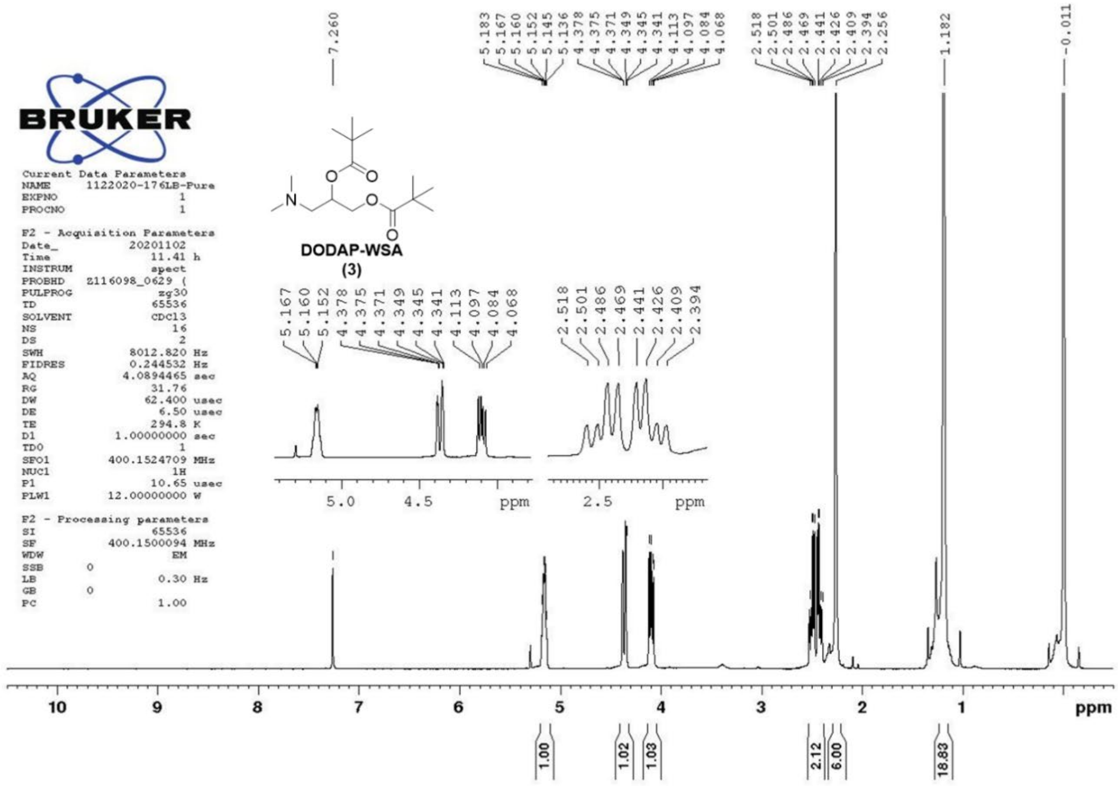

^{13}C & DEPT NMR of DODAP-WSA

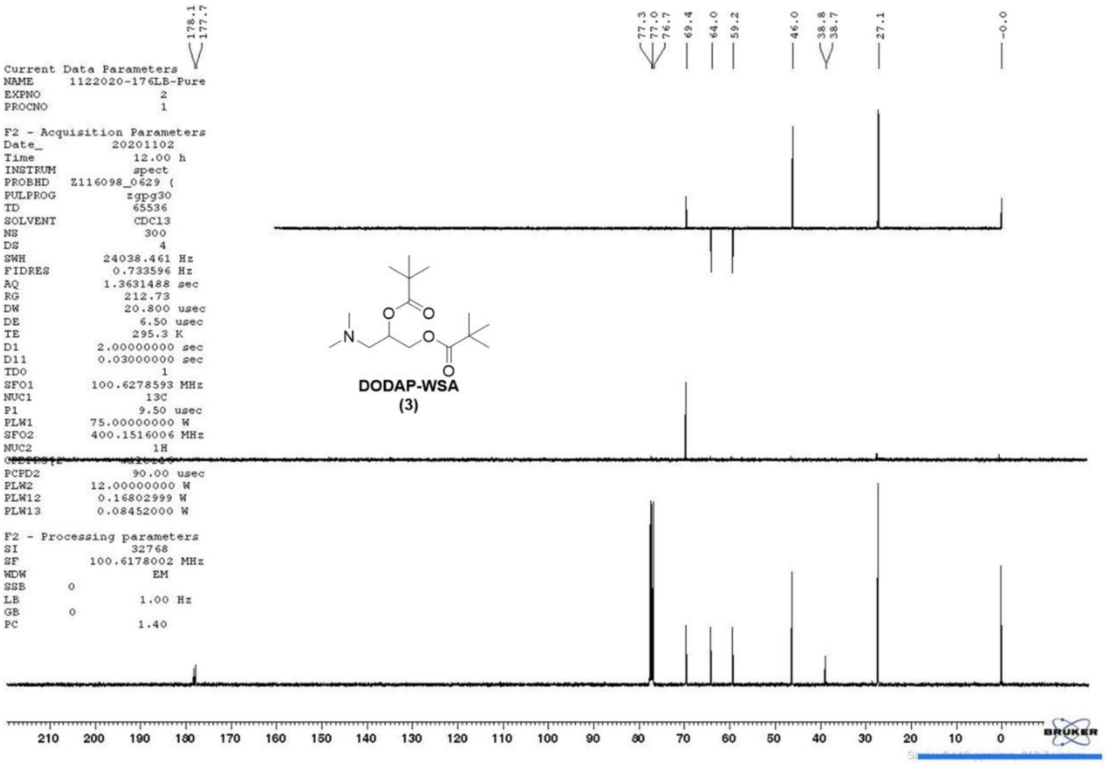

^{1}H NMR of DLin-WSA

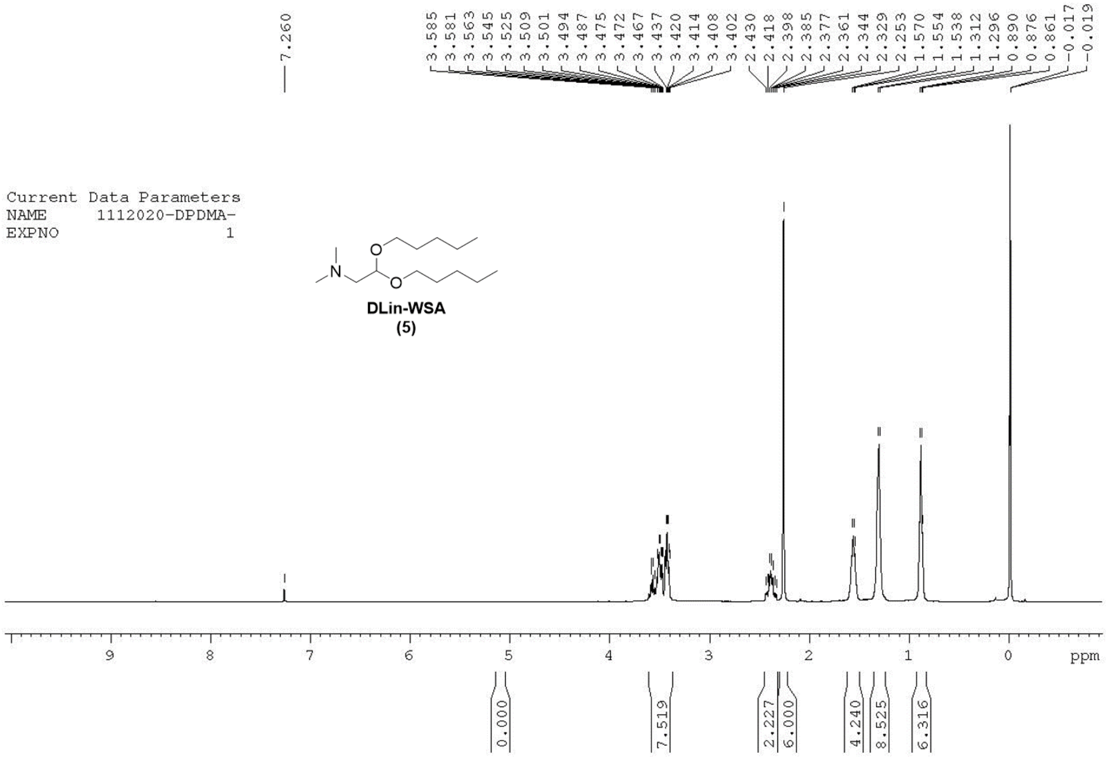

^{13}C & DEPT NMR of DLin-WSA

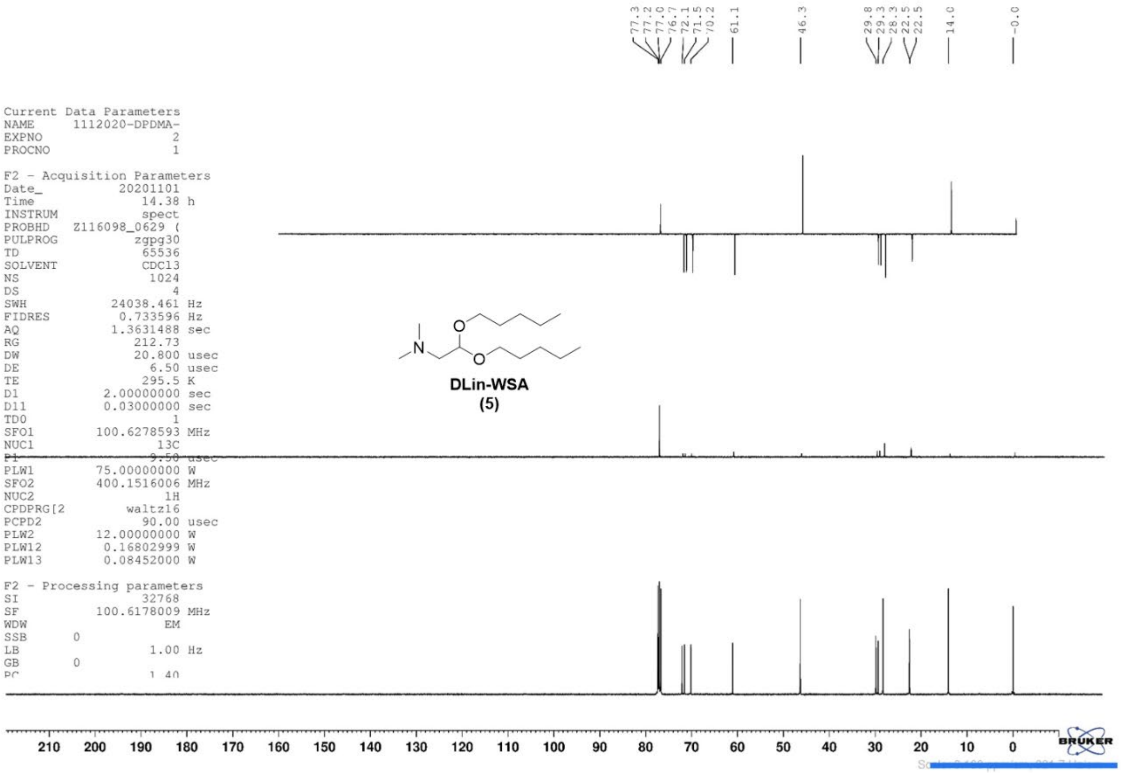

^{1}H NMR of MC3-WSA

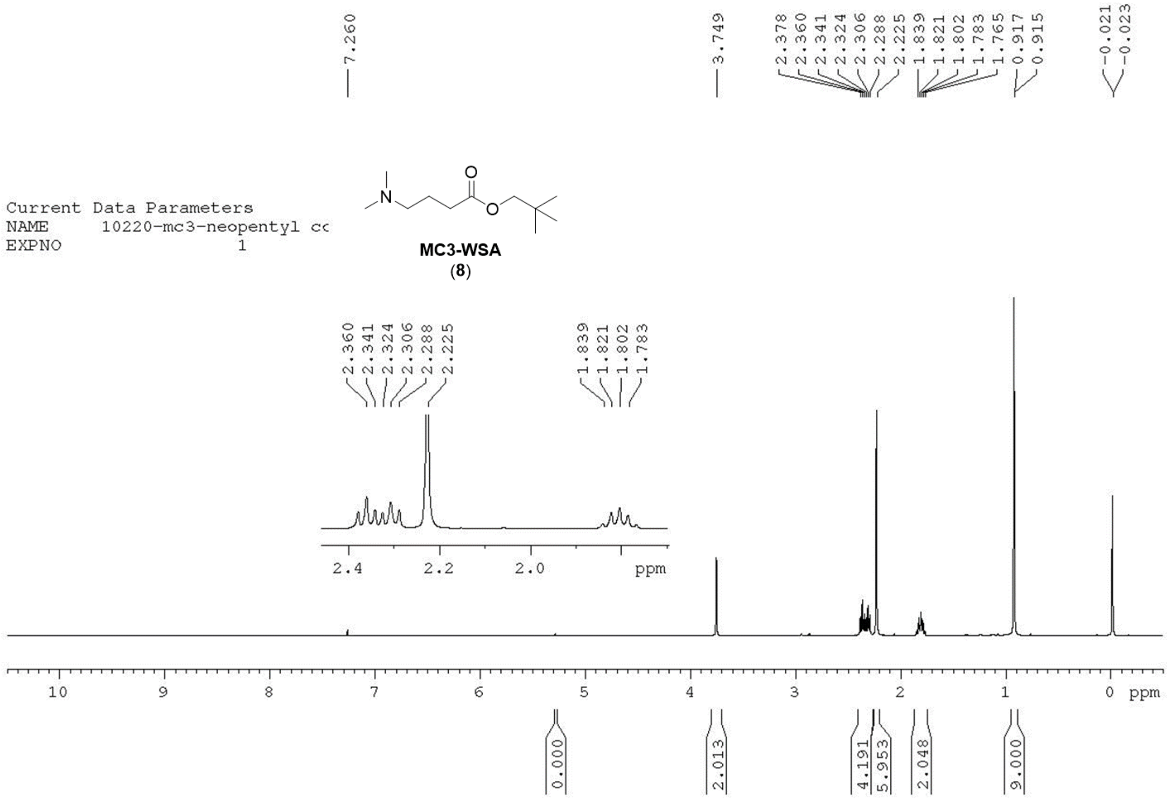

^{13}C & DEPT NMR of MC3-WSA

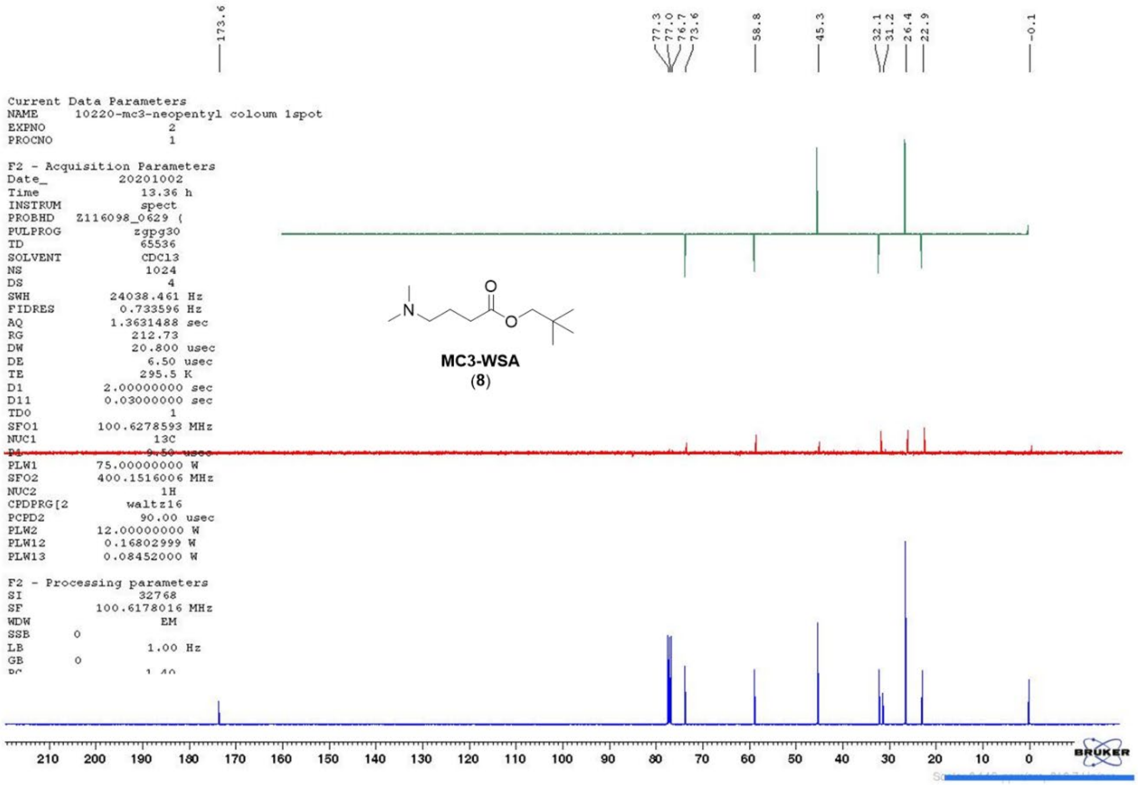

^{1}H NMR of KC2-WSA

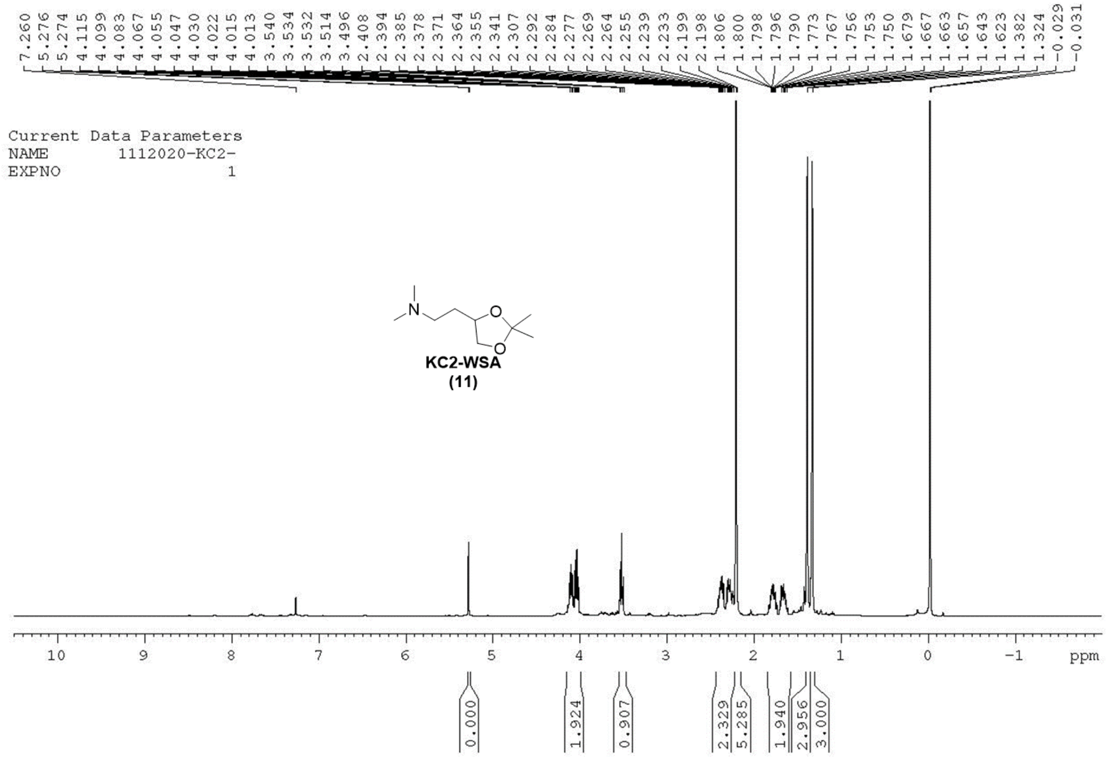

^{13}C& DEPT NMR of KC2-WSA

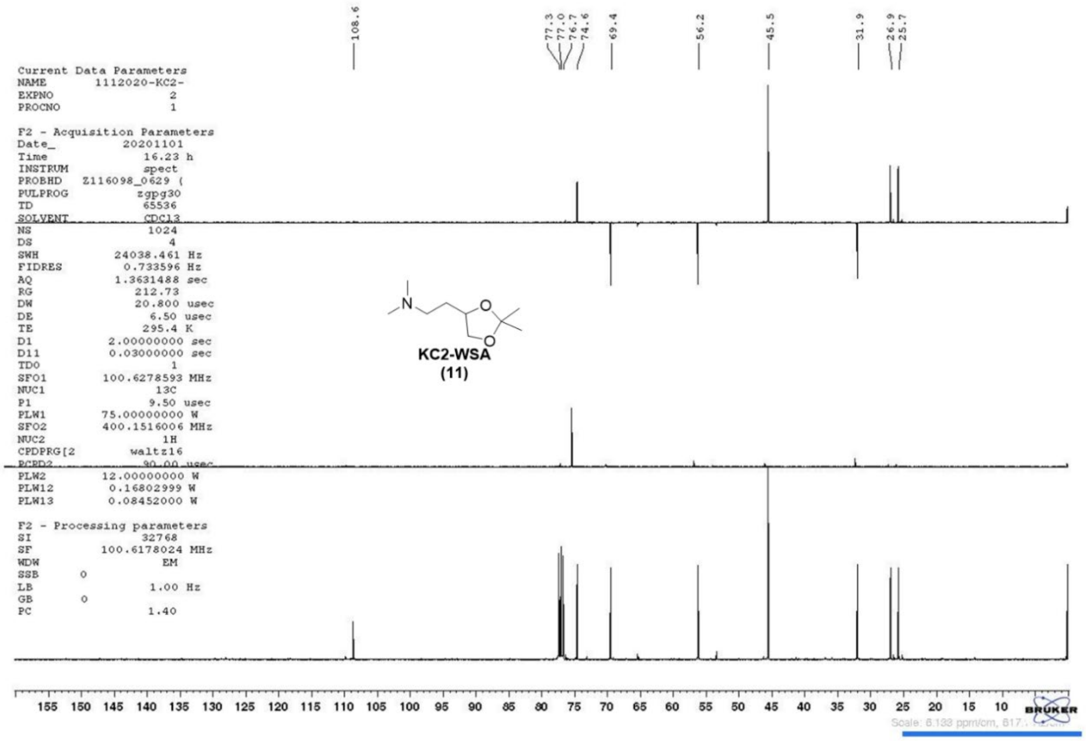

Supplementary Figure 5. ^{1}H, ^{13}C and DEPT NMR Data of Key Compounds

Calculation of pKa of the ionizable lipid in the LNP compared to the pKa of the ionizable lipid in the aqueous phase

Neglecting the proton activity coefficient, the electrochemical potential of the proton in the lipid nanoparticle (L) is

$$\mu_{L(H^+)} = \mu^{0L(H^+)} + kT\ln[H^+]_L + e\Psi_L \quad (1)$$

where $[H^+]_L$ is the proton concentration in the LNP, Ψ_L the electric potential of the LNP, and $\mu^{0L(H^+)}$ is the proton chemical potential in the LNP referred to a common reference when $[H^+]_L = 1M$ and $\Psi_L = 0$. Similarly, the proton electrochemical potential in the aqueous phase (W) is

$$\mu_{W(H^+)} = \mu^{0W(H^+)} + kT\ln[H^+]_W + e\Psi_W \quad (2)$$

Since the proton is in equilibrium between the LNP and the aqueous phase, $\mu_{L(H^+)} = \mu_{W(H^+)}$ resulting in

$$[H^+]_W = [H^+]_L e^{\frac{\mu^{0L(H^+)}-\mu^{0W(H^+)}}{kT}} e^{\frac{e(\Psi_L-\Psi_W)}{kT}} \quad (3)$$

The acid dissociation constant of the amine group of the ionizable lipid in the LNP is

$$K_{aL} = \frac{[N]_L[H^+]_L}{[NH^+]_L} \quad (4)$$

However, the pH is measured in the aqueous phase outside the LNP so that the measured pKa, or apparent pKa of the LNP measured by TNS binding assay or zeta potential is

$$K_{appL} = \frac{[N]_L[H^+]_W}{[NH^+]_L} \quad (5)$$

Substituting Eq 3 into Eq 5 results in

$$K_{appL} = \frac{[N]_L[H^+]_L}{[NH^+]_L} e^{\frac{\mu^{0L(H^+)}-\mu^{0W(H^+)}}{kT}} e^{\frac{ze(\Psi_L-\Psi_W)}{kT}} \quad (6)$$

or

$$K_{appL} = K_{aL} e^{\frac{\mu^{0L(H^+)} - \mu^{0W(H^+)}}{kT}} e^{\frac{ze(\Psi_L - \Psi_W)}{kT}} \quad (7)$$

such that

$$pK_{appL} = pK_{aL} - \frac{\mu^{0L(H^+)} - \mu^{0W(H^+)}}{2.303kT} - \frac{ze(\Psi_L - \Psi_W)}{2.303kT} \quad (8)$$

Recognizing $\mu^{0L(H^+)} - \mu^{0W(H^+)}$ as the energy of transfer of one proton from water to the LNP and taking the reference potential $\Psi_W = 0$, we write the above in molar units as

$$pK_{appL} = pK_{aL} - \frac{\Delta G_{tr}(H^+_{W \to L})}{2.303RT} - \frac{F\Psi_L}{2.303RT} \quad (9)$$

where $\Delta G_{tr}(H^+_{W \to L})$ is the molar free energy of transfer of protons from water to the lipid phase, F is Faraday's constant and R is the gas constant.

Eq 9 is the basis for understanding the measured 2-3 point drop in pKa of the ionizable lipid when it is in the LNP and measured by TNS binding assay and zeta potential, pK_{appL}, compared to when the ionizable lipid is in the aqueous phase, pK_a, measured by NMR on the water soluble analogue as predicted by ACDLabs *Percepta*. The main differences between the LNP medium and the aqueous medium for the ionization equilibrium are 1) the dielectric constant 2) the solvation energies of ionic species and 3) the electric potential. The dielectric constant of a lipid bilayer[11] is ~5 while our analyses of zeta potential data suggest an LNP dielectric constant of 6-24 that is consistent with the presence of some water in the LNP as found by neutron scattering analyses[12]. Water defects have also be predicted in molecular dynamics simulations of lipid bilayers[13]. Thus although some water is present in the LNP, its content is low such that the dielectric constant of the LNP is 6-24 rather than 80 for pure water or 5 for a lipid bilayer. This low water content and the nonpolar nature of the lipid phase render solvation energies of ionic species higher in the LNP versus water thereby partitioning protons to the water phase vs the lipid phase, effectively creating a pH drop from the lipid to water phase that is measured as an apparent pKa difference.

Considering each term on the right hand side of Eq 9 in order, we first take pK_{aL} which is the intrinsic pKa of the ionizable lipid in the lipid phase where the proton concentration cannot be measured. Since the ionization reaction $NH^+ \rightarrow N + H^+$ does not change the number of charged

species, the intrinsic pKa of the ionizable lipid will not be greatly different in the lipid versus the aqueous phase, due to medium dependent solvation energies or electrostatic energies. This is in contrast to the case of a molecular acid such as a carboxylic acid that increases the number of ionic species upon dissociation ($COOH \rightarrow COO^{-} + H^{+}$), generates electrostatic interactions, and the ionization products are favored in a polar medium with low solvation energies like water, shifting equilibrium to the right in water. If we consider the intrinsic pKa change for protonated anilines (analogous to the dimethylamine in the ionizable lipid) in methanol versus water their pKa in methanol is 0.7 units higher than in water ($pK_{aL} - pK_a$) due to a more easily solvated protonated aniline versus the proton itself in methanol vs water (Cox et al[14] Table 5.5 and Eq 5.6). If we now consider the second term in Eq 9, the free energy change of the proton from water to the lipid $\Delta G_{tr}(H^{+}_{W \rightarrow L})$ and again use methanol data to substitute for the lipid we find a 2 point drop in pKa due to the higher energy of solvation of the proton in methanol versus water (Cox et al[14] Table 3.5 : $\Delta G_{tr}(H^{+}_{W \rightarrow L}) = 10.4\ kJ/mol$). Thus combining the 2 point drop due to proton solvation (2nd term on the right in Eq 9) and 0.7 point increase due to the intrinsic pKa (1st term on the right in Eq 9), the net change from water to methanol is ~1.3 point drop, if we take methanol to represent the lipid nanoparticle and aniline to represent the ionizable lipid. However, this drop is underestimated since proton transfer energies from water to propanol and butanol for other ions such as sodium and potassium, where data is available, suggest higher 3 and 4 point drops in pKa due to the longer alkyl groups rendering protonation more difficult than in methanol, which is more similar to the lipid nanoparticle environment. Thus the combined effect of intrinsic pKa and proton solvation is likely to be a 2-3 point drop when placing the ionizable lipid in the LNP versus in water but still measuring the pH in the water phase. This interpretation is consistent with a previous study using ionizable fluorescent indicators associated with micelles[15] and explains our observed 2-3 point drop from the NMR measurements and ACDLabs predictions versus what is measured for LNPs using the TNS binding assay and ZP. Finally, the third term on the right in Eq 9, $F\Psi_L/2.303RT$, is proportional to the electric potential of the LNP which is positive at low pH lowering the pKa further and negative at high pH increasing the pKa. This electrostatic effect on the pKa is thus charge-dependent, similar to the ionization behaviour of a polyelectrolyte[16,17]. It becomes more energetically costly to protonate a positively charged LNP at low pH due to proton repulsion from the LNP and less energetically costly at high pH where protons are attracted to the LNP. This charge-dependent pKa of the LNP results in a much broader titration across ~4 pH units as seen

in Figs 1D and 6E for the Zeta Potential versus the expected ~2 pH units for a monoprotic acid and as seen for the NMR titrations (Fig 1A). Therefore, the zeta potential data is more accurately fit to the extended Henderson–Hasselbalch equation original proposed for polyelectrolytes[17], $pH = pKa - n\ln([N]/[NH^{+}])$, than the traditional Henderson–Hasselbalch equation, $pH = pKa - \ln([N]/[NH^{+}])$. TNS is only sensitive to initial protonation events at high pH since it interacts only with the surface of the LNP, so that the extended form of Henderson–Hasselbalch is not required to capture TNS titration behaviour.

Molecular Volume Model of the LNP estimates copies of mRNA per LNP and of the 4 lipid components

This molecular volume model calculates the number of each of the 5 constituents (4 lipids and mRNA) in the LNP, including the number of copies of mRNA in the LNP. The model assumes a spherical LNP, without any specific internal structure, and that each molecule of the 5 constituents occupies a volume in that sphere that is equal to their molecular volume reported in the literature and that the number of molecules of each constituent in the LNP respects the macroscopic mole fraction ratios. The N:P ratio, NP, is defined as the ratio of amine groups in the ionizable lipid to the phosphate groups on the mRNA backbone. The composition of the lipid mix in ethanol is described by the mole ratios of the 4 lipids in ethanol, the ionizable lipid with mole ratio in ethanol χ_{IE}, cholesterol with mole ratio in ethanol χ_{CE}, the helper lipid DSPC with mole ratio in ethanol χ_{HE}, and the pegylated lipid with mole ratio in ethanol χ_{PE}. The standard LNP formulation typically uses mole ratios 50:38.5:10:1.5 for χ_{IE}: χ_{CE}: χ_{HE}: χ_{PE}. Upon mixing with the nucleic acid the mole fractions of the 5 components become (without E subscript) :

$$\chi_I = \frac{\chi_{IE}}{\chi_{IE} + \chi_{IC} + \chi_{IH} + \chi_{IP} + {\chi_{IE}}/{NP}} \tag{10}$$

$$\chi_C = \frac{\chi_{CE}}{\chi_{IE} + \chi_{IC} + \chi_{IH} + \chi_{IP} + {\chi_{IE}}/{NP}} \tag{11}$$

$$\chi_H = \frac{\chi_{HE}}{\chi_{IE} + \chi_{IC} + \chi_{IH} + \chi_{IP} + {\chi_{IE}}/{NP}} \tag{12}$$

$$\chi_P = \frac{\chi_{PE}}{\chi_{IE} + \chi_{IC} + \chi_{IH} + \chi_{IP} + {\chi_{IE}}/{NP}} \tag{13}$$

$$\chi_N = \frac{{\chi_{IE}}/{NP}}{\chi_{IE} + \chi_{IC} + \chi_{IH} + \chi_{IP} + {\chi_{IE}}/{NP}} \tag{14}$$

where χ_N refers to the mole fraction of nucleotides. Molecular volumes in nm^3 of each of the constituents are taken from Table S1 in [12] :

$$V_{mI} = 1.29, V_{mC} = 0.63, V_{mH} = 1.32, V_{mP} = 0.67, V_{mN} = 0.325 \tag{15}$$

The average molecular volume of the mixture is

$$V_{mA} = \chi_I V_{mI} + \chi_C V_{mC} + \chi_H V_{mH} + \chi_P V_{mP} + \chi_N V_{mN} \tag{16}$$

The total number of molecules in the spherical volume of the LNP with diameter d is :

$$N_T = \frac{V_{LNP}}{V_{mA}}\left(1 - V_{fH_2O}\right) \tag{17}$$

where $V_{LNP} = \frac{4}{3}\pi\left(\frac{d}{2}\right)^3$ with d the number average diameter from DLS, and V_{fH_2O} is the fraction of the LNP volume occupied by water, that can be estimated at 25%[12]. The number of molecules of each of the 5 constituents can then be estimated as

$$N_I = \chi_I N_T;\ N_C = \chi_C N_T;\ N_H = \chi_H N_T;\ N_P = \chi_P N_T;\ N_N = \chi_N N_T; \tag{18}$$

It is assumed in the above that there is no significant free component of any constituent so that they are all found in the LNPs with the same mole fractions as in the macroscopic solution mixture. Since the encapsulation efficiency of mRNA is measured to be less than 100%, we can account for the fraction of mRNA in the LNP that is equal to the encapsulation efficiency EE, by replacing NP above with NP/EE which is the NP lipid:mRNA ratio within the LNP. Finally, the number of copies of mRNA in the LNP is :

$$N_{mRNA} = \frac{N_N}{N_{Bases}} \tag{19}$$

where N_{Bases} is the number of nucleotides in the mRNA sequence.

Calculation of Elementary Charge and Dielectric Constant of the LNP

Zeta potential measurements can provide an estimate of the number of elementary charges in the LNP if the LNP dielectric constant is known, or can provide the LNP dielectric constant if the LNP charge can be estimated. The zeta potential ζ in V (volts) is calculated from the measured electrophoretic mobility μ in $m^2/(\mathrm{V}\cdot\mathrm{s})$ using[18]

$$\zeta = \frac{3\eta\mu}{2\epsilon\epsilon_0 f(d/2\lambda)} \tag{20}$$

Where $\eta = 10.2 \times 10^{-4}\ \mathrm{Pa}\cdot\mathrm{s}$ is the viscosity of water at 20°C, $\epsilon = 80.2$ is the relative permittivity of water, $\epsilon_0 = 8.854 \times 10^{-12}\ \mathrm{Farad/m}$ is the permittivity of free space, f is Henry's function, d the diameter of the LNP and λ is the Debye length. The Debye length[19] in m as a function of the molar concentration c of mono-monovalent salt in $\mathrm{moles/L}$ is :

$$\lambda = \frac{A}{\sqrt{c}}\ ;\ \ A = \sqrt{\frac{\epsilon\epsilon_0 RT}{2000F^2}} \tag{21}$$

where $R = 8.3145\ \mathrm{J/(K\cdot mol)}$, $T = 298\ \mathrm{K}$ and $F = 96{,}485\ \mathrm{C/mol}$.

A convenient and accurate form of Henry's function[18] is

$$f(x) = \frac{3x^2 + 18x + 16}{2x^2 + 18x + 16} \tag{22}$$

The effective LNP surface charge in $\mathrm{C/m^2}$ that is consistent with the zeta potential according to the nonlinear Poisson-Boltzmann equation is[20] :

$$\sigma = \frac{\epsilon\epsilon_0 RT}{F\lambda}\left(2Sinh\left(\frac{\zeta F}{2RT}\right) + \frac{8\lambda}{d}Tanh\left(\frac{\zeta F}{4RT}\right)\right) \tag{23}$$

The number of elementary charges seen at the surface of the LNP is then :

$$Q_s = \pi d^2\sigma/e \tag{24}$$

where $e = 1.6022\ \times 10^{-19}\ \mathrm{C}$.

Finally, the net space charge distributed within the LNP arising from the anionic nucleic acid and the cationic ionizable lipid is within a mainly lipid domain of dielectric constant ϵ_{LNP} so that the number of elementary charges seen at the surface of the LNP is related to the actual number of charges in the LNP, Q_{LNP}, by

$$Q_s = Q_{LNP}/\,\epsilon_{LNP} \tag{25}$$

Q_s is then calculated directly from the measured zeta potential using Eqs 20-24 and Q_{LNP} or ϵ_{LNP} can be obtained from Eq 25 provided the other is given. For example LNP charge may be estimated at high pH where the ionizable lipid is neutral as Q_{LNP}=N_N calculated from the molecular volume model in the previous section, which then provides an estimate of the dielectric constant of the LNP using the measured Q_s as

$$\epsilon_{LNP} = N_N/Q_s \tag{26}$$

Materials and Methods

Materials

DLin-KC2-DMA was purchased from Biofine International INC. DLin-MC3-DMA was purchased from Advanced ChemBlock. DLin-DMA was purchased from Chem Scene. DMG-PEG (MW 2000) (DMG-PEG2000) was purchased from NOF America. Cholesterol was purchased from Combi Blocks. 1,2-dioleyloxy-3-dimethylaminopropane (DODMA), 1,2-dioleoyl-3-dimethylammonium-propane (18:1 DAP, DODAP) and 1,2-distearoyl-sn-glycero-3-phosphocholine (18:0 PC, DSPC), were purchased from Avanti Polar Lipids. Water-soluble analogues of DLin-KC2-DMA, DLin-MC3-DMA, DLin-DMA, DODMA and DODAP were synthetized and purified as described below. Microfluidic cartridges compatible with the Spark NanoAssmblr™ were purchased from Precision Nanosystems. 3M Sodium Acetate pH=5.5 was purchased from Thermofisher Scientific. Slide-A-Lyzer™ MINI Dialysis Device 0.5 ml (MWCO, 10 KDa) was purchased from Thermo Fisher Scientific. 6-(p-toluidino)-2-naphthalenesulfonic acid sodium salt (TNS) was purchased from Sigma. Codon optimized firefly luciferase (FLuc) sequence was cloned into an mRNA production plasmid (optimized 3' and 5' UTR and containing a 101 polyA tail), in vitro transcribed using N1-methylpseudouridine modified nucleoside, co-transcriptionally capped using the CleanCap technology (TriLink) and cellulose purified[21] to remove dsRNA. Purified mRNA was ethanol precipitated, washed, resuspended in nuclease-free water, and subjected to quality control (electrophoresis, dot blot, and transfection into human DCs).

Preparation of mRNA Lipid Nanoparticles

mRNA-loaded LNPs were formulated using total lipid concentration of 50 mM comprised of commercial ionizable lipids (DLin-KC2-DMA, DLin-MC3-DMA, DLin-DMA, DODMA, DODAP) /DSPC/Cholesterol/DMG-PEG2000 in the % mole ratios 50/10/38.5/1.5. Each lipid was dissolved in ethanol and mixed to reach the specified molar ratios in the organic phase. FLuc mRNA in the aqueous phase was dissolved in 43-50 mM sodium acetate buffer pH 4 to reach 1 mg/ml, keeping the NP lipid:RNA ratio (moles amine of the ionizable lipid: moles phosphate of the mRNA) constant at 4, prior to mixing in the Spark NanoAssmblr™ (Precision NanoSystems). A 16-µL aliquot of the organic phase and a 32-µL aliquot of the aqueous phase were mixed and ejected into 48 µL of PBS at pH 7.4. The LNPs were then diluted into an additional 96 µL DPBS solution at pH 7.4 and dialyzed against PBS to reach pH 7.3-7.4 after 4-6 buffer exchanges over 4-6 hours using a Slide-A-Lyzer MINI Dialysis Device (MWCO, 10 KDa). Empty LNPs were assembled with the lipid mole ratios 30-98.5:43.5-68.5:1.5 (DSPC:Cholesterol:PEG-DMG2000, and a total lipid mix of ~50 mM. Further procedures were performed as described above.

TNS Binding Assay

LNP pKa was determined using the TNS binding assay. The TNS reagent was prepared as a 300 µM stock solution in DMSO. Following Zhang et al 2011[4], LNPs were diluted to 75 µM ionizable lipid, TNS to 6 µM in a total volume of ~93 µL of buffered solutions containing 20 mM boric Acid, 10 mM imidazole, 10 mM sodium acetate, 10 mM glycylglycine, 25 mM NaCl, where the pH ranged from 3 to 10. The Cytation 5 Cell Imaging Multi-Mode Reader (Biotek) was used to read Fluorescence (Ex321/Em445). The pH was measured in each well after TNS addition. Mathematica (Wolfram Research) was used to fit the fluorescence data to the Henderson–

Hasselbalch equation $\mathrm{RFU} = \mathrm{RFU}_{max} - (\mathrm{RFU}_{max} - \mathrm{RFU}_{min})/(1 + 10^{pKa-pH})$ to provide the pKa. We also tested two alternative TNS binding assay protocols (Supp. Figures 1 & 2, Supp. Table 1 & 2) Sabnis et al 2018[5], where LNPs were diluted to 24 µM and TNS to 6.3 µM, and Jayaraman et al 2012[2], where LNPs were diluted to 40 µM and TNS to 1 µM.

Empty LNP size and zeta potential (ZP) using dynamic light scattering and electrophoretic mobility

LNPs were diluted to 6.25 ng/µL total mRNA in PBS pH=7.4 and transferred into a quartz cuvette (ZEN2112) to measure size by Dynamic Light Scattering (DLS) in the Zetasizer Nano ZS (Malvern Panalytical) using particle RI of 1.45 and absorption of 0.001 in PBS at 25 °C with viscosity of 0.888 cP and RI of 1.335. Measurements were made using a 173° backscatter angle of detection previously equilibrated to 25 °C for 30 seconds in duplicates, each with 5 runs and 10 second run duration, without delay between measurements. Each measurement had a fixed position of 4.65 mm in the quartz cuvette with an automatic attenuation selection. Data was analysed using a General-Purpose model with normal resolution. Diameter are reported as the number-average. LNPs were diluted into the TNS buffer described above at pH ranging from 3-10 for zeta potential measurement by Electrophoretic Light Scattering (ELS) in the Zetasizer Nano ZP (Malvern Panalytical) using the same material and dispersant parameters described above, and the Smoluchowski model. Each measurement had voltage set manually at 80 Volts to avoid ohmic heating that occurred if voltage was set automatically. Measurements in the disposable folded capillary cuvette with an automatic attenuation selection for photon counts were made in triplicates for 20 runs each, and 30 second delay between each replicate.

Synthesis of water-soluble ionizable lipid analogues

Solvents were purchased from Sigma Aldrich, Combi blocks, Oakwood chemicals, Alfa Aesar, VWR and Thermofisher. Anhydrous methylene chloride (DCM), anhydrous tetrahydrofuran (THF) anhydrous DMF were purchased from Sigma Aldrich. Product purification was done using SiliaFlash® Irregular Silica Gel, F60 40 - 63 µm, 60 Å and SiliaPlate™ thin layer chromatography Plates (TLC), Glass-Backed Silica, Opt. $KMnO_4$, 250 µm, 20 x 20 cm, F254 plates purchased from Silicycle. NMR spectra were recorded on a Bruker 400 MHz Spectrometers using $CDCl_3$, D_2O (Sigma Aldrich), as d-solvents and internal standards (δ 7.26 for ^1H NMR and δ 77.00 for ^{13}C NMR). Solution of 1 M NaOH and 1 M HCl were purchased from Sigma Aldrich.

a) DODAP water-soluble analogue

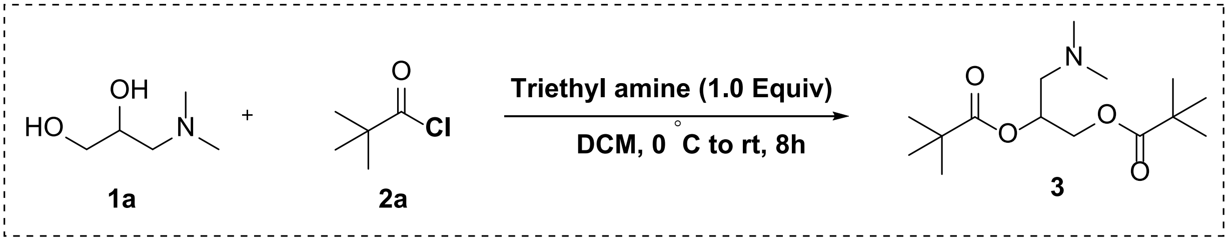

DODAP water-soluble analogue (**3** above) was synthesized as per the previous reports[22,23]. 3-(dimethylamino)propane-1,2-diol (**1a)** (16.8 mmol, 2.0 g, 1.99 mL), triethylamine (42 mmol, 4.25 g, 5.83 mL), and CH_2Cl_2 (200 mL) were placed in a 250-mL two-neck round bottom flask and cooled to 0 °C in an ice bath. The reaction was stirred for 1 h and then pivaloyl chloride (**2a)** (37.8 mmol, 4.55 g, 4.65 mL) was added dropwise and the reaction stirred for 7 h. The reaction progress was monitored every 3 h by TLC (chloroform/methanol 9:1 v/v, can be visualized with iodine stain). After complete consumption of 1a, the reaction was quenched by adding water. CH_2Cl_2 was concentrated by rotary evaporation. The mixture was dissolved in 200 mL of CH_2Cl_2 and washed

with 150 mL of water and 150 mL of saturated $NaHCO_3$ solution. The organic phase was dried over magnesium sulphate and evaporated. The crude product was purified on a silica gel column eluted with chloroform containing 0-2% methanol. Column fractions were analyzed by thin layer chromatography (TLC) and fractions containing pure product (R_f=0.5) were pooled and concentrated, to obtain the product as yellow oil (**3**) (3.56 g, 62% yield). HNMR data (400 MHz, $CDCl_3$, δ = 7.26 ppm as standard): δ 5.17-5.15 (m, 1H), 4.38-4.34 (m, 1H), 4.09 (dd, 6.44 Hz, 6.44 Hz, 1H), 2.45 (dddd, 6.68 Hz, 6.68 Hz, 5.92 Hz, 5.80 Hz, 2H), 2.26 (s, 6H), 1.18 (s, 18H). ^{13}C NMR: (100 MHz, $CDCl_3$, δ = 77.0 ppm as standard): δ 178.1 (C), 177.7 (C), 69.4 (CH), 64.0 (CH_2), 59.2 (CH_2), 46.0 (2 × CH_3), 38.8 (C), 38.7 (C), 27.1 (6 × CH_3).

b) DLin-DMA/DODMA water-soluble analogue

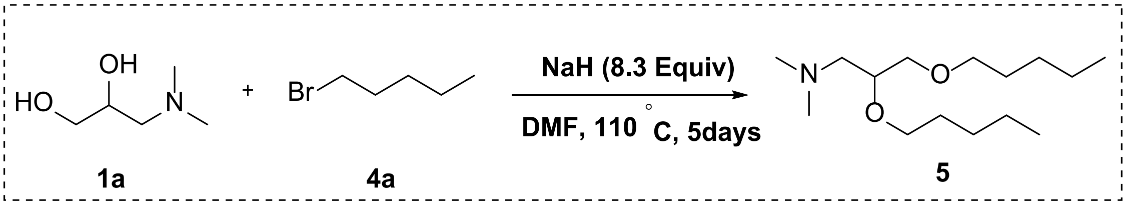

DLinDMA/DODMA water soluble analogue (**5** above) was synthesized as per the previous reports[6,24–26]. Excess sodium hydride NaH (8.3 equiv, 140 mmol, 3.34 g, 60% in oil) was added into a 250-mL two-neck round bottom (RB) flask under nitrogen atmosphere and anhydrous DMF (10 mL) was added. The resulting suspension was stirred for 10 minutes. To this slurry, a solution of 3-(dimethylamino)-propane-1,2-diol (**1a)** (1.0 equiv, 16.8 mmol, 2.0 g) in DMF (15 mL) was added dropwise for 10 minutes at 0 ºC. The resulting suspension was heated to reflux for 24-36 h. Pentyl bromide (**4a)** (2.50 equiv, 42.0 mmol, 5.2 ml) in DMF (10 mL) solution was prepared and added to the reaction mixture at 0 ºC. After addition, the reaction was heated to reflux for 5 days and then cooled to room temperature and the mixture filtered through a 3-cm plug of celite and

washed with methylene chloride (200 mL). The filtrate was removed under vacuum and the residue was partitioned between methylene chloride (100 mL), and brine (50 mL), used to aid phase separation. The two layers were separated and the organic layer was washed with dried over anhydrous magnesium sulfate. The crude product was purified on a silica gel column eluted with chloroform containing 0-5% methanol. Column fractions were analyzed by thin layer chromatography (TLC) (silica gel, chloroform/methanol 9:1 v/v, can be visualized with iodine or PMB stain) and fractions containing pure product (R_f=0.5) were pooled and concentrated, to obtain product as brown oil (**5**) (1.92 g, 37% yield). ^{1}H-NMR : (400 MHz, $CDCl_3$, δ = 7.26 ppm as standard): δ 3.58-3.54 (m, 1H), 3.52-3.47 (m, 2H), 3.44-3.40 (m, 4H), 2.43-2.33 (m, 2H), 2.25 (s, 6H, 2×NCH_3), 1.55 (t, 6.34 Hz, 4H), 1.31-1.29 (m, 8H), 0.89-0.86 (m, 6H), 0.86 (t, 6H, 2×CH_3). ^{13}C NMR: (100 MHz, $CDCl_3$, δ = 77.0 ppm as standard): δ 77.2 (C), 72.1 (CH_2), 71.5 (CH_2), 70.2 (CH_2), 61.1 (CH_2), 46.3 (2 × CH_3), 29.8 (CH_2), 29.3 (CH_2), 28.3 (2 × CH_2), 22.5 (2 × CH_2), 14.0 (2 × CH_3).

c) DLin-MC3-DMA water-soluble analogue

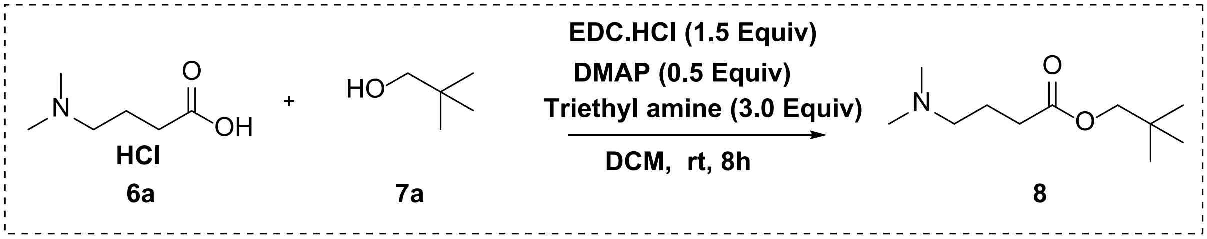

DLin-MC3-DMA water soluble analogue (**8** above) was synthesized as per the previous literature reports[27,28]. A mixture of 4-(dimethylamino)butanoic acid hydrochloride salt (**6a)** (59.7 mmol, 1.0 g), 1-(3-dimethylaminopropyl)-3-ethylcarbodiimide hydrochloride (EDCi•HCl) (7.16 mmol, 0.63 g), 4-(dimethylamino)pyridine (DMAP) (1.49 mmol, 0.18 g), triethylamine (42.0 mmol, 4.25 g, 5.83 mL), and CH_2Cl_2 (100 mL) were placed in a 250-mL two-neck round bottom flask. The

reaction was stirred for 20 minutes at room temperature and neopentyl alcohol (**7a)** (7.16 mmol, 0.78 mL) was added dropwise and the reaction stirred overnight. Reaction progress was monitored by TLC (chloroform/methanol 9:1 v/v, can be visualized with iodine stain), after complete consumption of **6a** the solvent CH_2Cl_2 was removed by rotary evaporation. The mixture was dissolved in 100 mL of CH_2Cl_2 and washed with 150 mL of water and 150 mL of saturated $NaHCO_3$ solution. The organic phase was dried over magnesium sulphate and evaporated. The crude product was purified on a silica gel column eluted with chloroform containing 0-1% methanol. Column fractions were analyzed by thin layer chromatography (TLC) and fractions containing pure product (R_f=0.4) were concentrated, to obtain product as yellow oil (**8**). (0.58 g, 48% yield). ^{1}H-NMR : (400 MHz, $CDCl_3$, δ = 7.26 ppm as standard): δ 2.36 (t, 7.4 Hz, 2H), 2.31 (t, 7.2 Hz, 2H), 2.22 (s, 6H, 2×NCH_3), 1.84-1.78 (m, 2H), 0.86 (s, 9H, 2×CH_3). ^{13}C NMR: (100 MHz, $CDCl_3$, δ = 77.0 ppm as standard): δ 173.6 (C), 73.6 (CH_2), 58.8 (CH_2), 45.3 (2 × CH_3), 32.1 (CH_2), 31.2 (C), 26.4 (9 × CH_3), 22.9 (2 × CH_2).

d) DLin-KC2-DMA water-soluble analogue

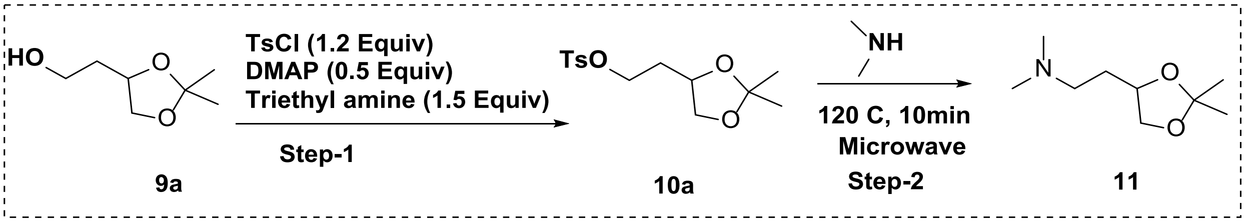

Step-1: DLin-KC2-DMA water soluble analogue (**11** above) was synthesized as per previous literature reports[29,30]. To a solution of 2-(2,2-dimethyl-1,3-dioxolan-4-yl)ethanol (**9a**) (800 mg, 5.47 mmol) in methylene chloride (25 mL) in a 1000-mL RBF at cooled to 0 °C was added triethylamine (0.915 mL, 6.57 mmol), 4-(dimethylamino)pyridine (DMAP) (134 mg, 1.10 mmol), and tosyl chloride (1.10 g, 5.75 mmol). The reaction was stirred at room temperature overnight.

The reaction was dried over Na_2SO_4 and concentrated. Ethyl ether 50ml was added to the crude product and the filtrate was concentrated to obtain colorless oil. The compound was used directly to the next step without purification (**10a**) (1.06 g, 65%).

Step-2: A Biotage microwave vial was equipped with a stir bar, 2-(2,2-dimethyl-1,3-dioxolan-4-yl)ethyl 4-methylbenzenesulfonate (**10a**) (500 mg, 1.66 mmol), and 1.0 M diethylamine in methanol (4 mL). The vial was purged with N_2 gas, seal capped, and heated at 120 °C for 10 minutes in a Biotage Initiator microwave. The reaction was diluted with ethyl acetate and water. The layers were separated and the aqueous layer was extracted (30 mL) with ethyl acetate. The combined organics were dried over Na_2SO_4, filtered and concentrated by rotary evaporation. The residue was of sufficient purity for determination of pKa by NMR without further purification. (**11**) (0.21 g, 75%).^{1}H-NMR : (400 MHz, $CDCl_3$, δ = 7.26 ppm as standard): δ 4.15-4.01 (m, 2H), 3.52 (t, 7.2 Hz, 2H), 2.41-2.23 (m, 2H), 2.20 (s, 6H, 2×NCH_3), 1.81-1.62 (m, 2H), 1.38 (s, 3H, CH_3), 1.32 (s, 3H, CH_3). ^{13}C NMR: (100 MHz, $CDCl_3$, δ = 77.0 ppm as standard): δ 108.6 (C), 74.6 (CH), 69.4 (CH_2), 56.2 (CH_2), 45.5 (2 × CH_3), 31.9 (CH_2), 26.9 (CH_3), 25.7 (CH_3).

NMR measurement of pKa of water-soluble ionizable lipid analogues

The pH-dependence of proton NMR chemical shifts was used to measure the pKas of the ionizable lipid water-soluble analogues following published methods[1,31]. Chemical shifts of piperazine and imidazole were used as internal pH indicators. Solutions were prepared with 100 mM KCl, 2 mM piperazine, 2 mM imidazole, and 5 mM water soluble ionizable lipid analogue in 95% H_2O-5% D_2O. This solution was split into two equal volumes and one titrated to a lower pH (e.g. 6) using 0.1 M HCl and the other to an upper pH (e.g. 12) using 0.1 M NaOH. Intermediate pH values were obtained by mixing different proportions of these two solutions. NMR measurements were

performed on a Bruker 400 MHz spectrometer where ^1H spectra were acquired at each of ~20 pH values ranging from the lower to upper pH (e.g. 6-12). Chemical shifts from piperazine and imidazole were then used to calculate the pH of each solution according to published methods[1,31] and the chemical shifts of the N-terminal amine group protons of the water-soluble ionizable lipid analogues were fit to the Henderson-Hasselbalch equation $\delta = \delta_{max} - (\delta_{max} - \delta_{min})/(1 + 10^{pKa-pH})$ to provide the pKa of the N,N-Dimethylamine moiety in the different head groups (Spectra in Supp Figure 1).

Supplementary References

(1) Oregioni, A.; Stieglitz, B.; Kelly, G.; Rittinger, K.; Frenkiel, T. Determination of the PK a of the N-Terminal Amino Group of Ubiquitin by NMR. *Sci. Rep.* **2017**, *7* (1), 1–8. https://doi.org/10.1038/srep43748.

(2) Jayaraman, M.; Ansell, S. M.; Mui, B. L.; Tam, Y. K.; Chen, J.; Du, X.; Butler, D.; Eltepu, L.; Matsuda, S.; Narayanannair, J. K.; Rajeev, K. G.; Hafez, I. M.; Akinc, A.; Maier, M. A.; Tracy, M. A.; Cullis, P. R.; Madden, T. D.; Manoharan, M.; Hope, M. J. Maximizing the Potency of SiRNA Lipid Nanoparticles for Hepatic Gene Silencing in Vivo. *Angew. Chem. Int. Ed Engl.* **2012**, *51* (34), 8529–8533. https://doi.org/10.1002/anie.201203263.

(3) Semple, S. C.; Akinc, A.; Chen, J.; Sandhu, A. P.; Mui, B. L.; Cho, C. K.; Sah, D. W. Y.; Stebbing, D.; Crosley, E. J.; Yaworski, E.; Hafez, I. M.; Dorkin, J. R.; Qin, J.; Lam, K.; Rajeev, K. G.; Wong, K. F.; Jeffs, L. B.; Nechev, L.; Eisenhardt, M. L.; Jayaraman, M.; Kazem, M.; Maier, M. A.; Srinivasulu, M.; Weinstein, M. J.; Chen, Q.; Alvarez, R.; Barros, S. A.; De, S.; Klimuk, S. K.; Borland, T.; Kosovrasti, V.; Cantley, W. L.; Tam, Y. K.; Manoharan, M.; Ciufolini, M. A.; Tracy, M. A.; de Fougerolles, A.; MacLachlan, I.; Cullis, P. R.; Madden, T. D.; Hope, M. J. Rational Design of Cationic Lipids for SiRNA Delivery. *Nat. Biotechnol.* **2010**, *28* (2), 172–176. https://doi.org/10.1038/nbt.1602.

(4) Zhang, J.; Fan, H.; Levorse, D. A.; Crocker, L. S. Ionization Behavior of Amino Lipids for SiRNA Delivery: Determination of Ionization Constants, SAR, and the Impact of Lipid PKa on Cationic Lipid−Biomembrane Interactions. *Langmuir* **2011**, *27* (5), 1907–1914. https://doi.org/10.1021/la104590k.

(5) Sabnis, S.; Kumarasinghe, E. S.; Salerno, T.; Mihai, C.; Ketova, T.; Senn, J. J.; Lynn, A.; Bulychev, A.; McFadyen, I.; Chan, J.; Almarsson, Ö.; Stanton, M. G.; Benenato, K. E. A Novel Amino Lipid Series for MRNA Delivery: Improved Endosomal Escape and Sustained Pharmacology and Safety in Non-Human Primates. *Mol. Ther.* **2018**, *26* (6), 1509–1519. https://doi.org/10.1016/j.ymthe.2018.03.010.

(6) Heyes, J.; Palmer, L.; Bremner, K.; MacLachlan, I. Cationic Lipid Saturation Influences Intracellular Delivery of Encapsulated Nucleic Acids. *J. Controlled Release* **2005**, *107* (2), 276–287. https://doi.org/10.1016/j.jconrel.2005.06.014.

(7) Hajj, K. A.; Ball, R. L.; Deluty, S. B.; Singh, S. R.; Strelkova, D.; Knapp, C. M.; Whitehead, K. A. Branched-Tail Lipid Nanoparticles Potently Deliver MRNA In Vivo Due to Enhanced Ionization at Endosomal PH. *Small* **2019**, *15* (6), 1805097. https://doi.org/10.1002/smll.201805097.

(8) Semple, S. C.; Klimuk, S. K.; Harasym, T. O.; Dos Santos, N.; Ansell, S. M.; Wong, K. F.; Maurer, N.; Stark, H.; Cullis, P. R.; Hope, M. J.; Scherrer, P. Efficient Encapsulation of Antisense Oligonucleotides in Lipid Vesicles Using Ionizable Aminolipids: Formation of Novel Small Multilamellar Vesicle Structures. *Biochim. Biophys. Acta BBA - Biomembr.* **2001**, *1510* (1), 152–166. https://doi.org/10.1016/S0005-2736(00)00343-6.

(9) Zimmermann, R.; Freudenberg, U.; Schweiß, R.; Küttner, D.; Werner, C. Hydroxide and Hydronium Ion Adsorption — A Survey. *Curr. Opin. Colloid Interface Sci.* **2010**, *15* (3), 196–202. https://doi.org/10.1016/j.cocis.2010.01.002.

(10) Klasczyk, B.; Knecht, V.; Lipowsky, R.; Dimova, R. Interactions of Alkali Metal Chlorides with Phosphatidylcholine Vesicles. *Langmuir* **2010**, *26* (24), 18951–18958. https://doi.org/10.1021/la103631y.

(11) Weaver, J. C.; Schoenbach, K. H. Biodielectrics. *IEEE Trans. Dielectr. Electr. Insul.* **2003**, *10* (5), 715–716. https://doi.org/10.1109/TDEI.2003.1237322.

(12) Yanez Arteta, M.; Kjellman, T.; Bartesaghi, S.; Wallin, S.; Wu, X.; Kvist, A. J.; Dabkowska, A.; Székely, N.; Radulescu, A.; Bergenholtz, J.; Lindfors, L. Successful Reprogramming of Cellular Protein Production through MRNA Delivered by Functionalized Lipid Nanoparticles. *Proc. Natl. Acad. Sci.* **2018**, *115* (15), E3351–E3360. https://doi.org/10.1073/pnas.1720542115.

(13) Yoo, J.; Cui, Q. Chemical versus Mechanical Perturbations on the Protonation State of Arginine in Complex Lipid Membranes: Insights from Microscopic PKa Calculations. *Biophys. J.* **2010**, *99* (5), 1529–1538. https://doi.org/10.1016/j.bpj.2010.06.048.

(14) Cox, B. G. *Acids and Bases: Solvent Effects on Acid-Base Strength*; OUP Oxford, 2013.

(15) Fernandez, M. S.; Fromherz, P. Lipoid PH Indicators as Probes of Electrical Potential and Polarity in Micelles. *J. Phys. Chem.* **1977**, *81* (18), 1755–1761. https://doi.org/10.1021/j100533a009.

(16) Filion, D.; Lavertu, M.; Buschmann, M. D. Ionization and Solubility of Chitosan Solutions Related to Thermosensitive Chitosan/Glycerol-Phosphate Systems. *Biomacromolecules* **2007**, *8* (10), 3224–3234. https://doi.org/10.1021/bm700520m.

(17) Katchalsky, A.; Spitnik, P. Potentiometric Titrations of Polymethacrylic Acid. *J. Polym. Sci.* **1947**, *2* (4), 432–446. https://doi.org/10.1002/pol.1947.120020409.

(18) Swan, J. W.; Furst, E. M. A Simpler Expression for Henry's Function Describing the Electrophoretic Mobility of Spherical Colloids. *J. Colloid Interface Sci.* **2012**, *388* (1), 92–94. https://doi.org/10.1016/j.jcis.2012.08.026.

(19) Dill, K.; Bromberg, S. *Molecular Driving Forces: Statistical Thermodynamics in Biology, Chemistry, Physics, and Nanoscience*; Garland Science, 2012.

(20) Doane, T. L.; Chuang, C.-H.; Hill, R. J.; Burda, C. Nanoparticle ζ -Potentials. *Acc. Chem. Res.* **2012**, *45* (3), 317–326. https://doi.org/10.1021/ar200113c.

(21) Baiersdörfer, M.; Boros, G.; Muramatsu, H.; Mahiny, A.; Vlatkovic, I.; Sahin, U.; Karikó, K. A Facile Method for the Removal of DsRNA Contaminant from In Vitro-Transcribed MRNA. *Mol. Ther. - Nucleic Acids* **2019**, *15*, 26–35. https://doi.org/10.1016/j.omtn.2019.02.018.

(22) Kumar, P.; ELSAIDI, H.; Wiebe, L. I.; Weinfeld, M.; RICARDO, C. L. Bioreductively-Activated Compounds, Their Prodrugs, Radiopharmaceuticals, the Compositions, and Their Applications in Multimodal Theranostic Management of Hypoxia Diseases Including Cancer. WO2019056098A1, March 28, 2019.

(23) Smith, P. G.; Dhanuka, V. V.; Hwang, H. S.; Lim, K. T.; Johnston, K. P. Tertiary Amine Esters for Carbon Dioxide Based Emulsions. *Ind. Eng. Chem. Res.* **2007**, *46* (8), 2473–2480. https://doi.org/10.1021/ie060934h.

(24) Bennett, M. J.; Boylan, J. F.; Goodnow, R. A.; He, W.; Lin, T.; Sidduri, A. New (S)-2-(Cyclopentanecarbonyl-Amino)-3-Phenyl-Propionic Acid Compounds Are Very Late Antigen-4 Inhibitors, Useful for e.g. Treating Cancer or Inflammatory Diseases. US 20130079383 A1, 2013.

(25) Hu, X.; Liu, M.; Liu, S.; Su, S.; Xiang, S.; Zeng, Y.; Zhang, J.; Zhao, C. Propylene Glycol Amine Derivative of Cationic Liposome Nanoparticle to Deliver Nucleic Acid Comprises Iodized 2,3-Dialkoxyl-1-(N,N,N-Trimethyl)Propylamine, or Brominated 2,3-Dialkoxyl-1-(N,N-Dimethyl-N-(2-Ethoxy)Propylamine Nanoparticle. CN 102525926 A, 2011.

(26) Felgner, P. L.; Gadek, T. R.; Holm, M.; Roman, R.; Chan, H. W.; Wenz, M.; Northrop, J. P.; Ringold, G. M.; Danielsen, M. Lipofection: A Highly Efficient, Lipid-Mediated DNA-Transfection Procedure. *Proc. Natl. Acad. Sci.* **1987**, *84* (21), 7413–7417. https://doi.org/10.1073/pnas.84.21.7413.

(27) Sartoru, M.; Yoshimasa, O.; Masahiro, M.; Yasatuka, H.; Matsumoto, S.; Omori, Y.; Mineno, M.; Hoashi, Y. New Dimethylamino-Alkanoyloxy-Bis-((Tetradec-9-Enoyloxy)Methyl)Propyl Tetradec-9-Enoate Derivative Useful in Composition for Introducing Nucleic Acid e.g. Small Interfering RNA for Treating Disease e.g. Anemia, Psoriasis, and Lung Cancer. WO2019131839-A1, 2019.

(28) Liang, X.; Li, X.; Jing, L.; Xue, P.; Jiang, L.; Ren, Q.; Dai, Z. Design and Synthesis of Lipidic Organoalkoxysilanes for the Self-Assembly of Liposomal Nanohybrid Cerasomes with Controlled Drug Release Properties. *Chem. – Eur. J.* **2013**, *19* (47), 16113–16121. https://doi.org/10.1002/chem.201302518.

(29) Baryza, J.; Bowman, K.; Geall, A.; Labonte, T.; Lee, C.; Vargeese, C.; West, L.; Zhao, J. Lipids, Lipid Compositions, and Methods of Using Them. WO2011076807A2, June 30, 2011.

(30) Dande, P. A.; Hansen, T. M.; Hubbard, R. D.; Sarthy, A. V.; Shen, Y.; Tian, L.; Wada, C. K.; Zhao, X. Cationic Lipids and Uses Thereof. US20100055169A1, March 4, 2010.

(31) Baryshnikova, O. K.; Williams, T. C.; Sykes, B. D. Internal PH Indicators for Biomolecular NMR. *J. Biomol. NMR* **2008**, *41* (1), 5–7. https://doi.org/10.1007/s10858-008-9234-6.
