# Supplementary material for: Ionization and structural properties of mRNA lipid nanoparticles influence expression in intramuscular and intravascular administration
Source: Commun Biol. 2021 Aug 11;4:956. doi: 10.1038/s42003-021-02441-2 (PMC8358000; doi:10.1038/s42003-021-02441-2)
Supplement: Supplementary file 3 — Description of Additional Supplementary Files [file 42003_2021_2441_MOESM3_ESM.pdf]

Description of Additional Supplementary Files

File name: Supplementary Data 1

Description: Raw data for Figure 1

File name: Supplementary Data 2

Description: Raw data for Figure 2

File name: Supplementary Data 3

Description: Raw data for Figure 3

File name: Supplementary Data 4

Description: Raw data for Figure 4

File name: Supplementary Data 5

Description: Raw data for Figure 5

File name: Supplementary Data 6

Description: Raw data for Figure 6

File name: Supplementary Data 7

Description: Raw data for Figure 7
